# EGFR and EGFRvIII Promote Angiogenesis and Cell Invasion in Glioblastoma: Combination Therapies for an Effective Treatment

**DOI:** 10.3390/ijms18061295

**Published:** 2017-06-18

**Authors:** Stefanie Keller, Mirko H. H. Schmidt

**Affiliations:** 1 Molecular Signal Transduction Laboratories, Institute for Microscopic Anatomy and Neurobiology, Focus Program Translational Neuroscience (FTN), Rhine Mainz Neuroscience Network (rmn2), School of Medicine, Johannes Gutenberg University, 55131 Mainz, Germany; stefanie.keller@unimedizin-mainz.de; 2 German Cancer Consortium (DKTK), partner site Frankfurt/Mainz, 55131 Mainz, Germany; 3 German Cancer Research Center (DKFZ), 69120 Heidelberg, Germany

**Keywords:** glioblastoma multiforme, invasion, angiogenesis, EGFR, EGFRvIII

## Abstract

Epidermal growth factor receptor (EGFR) and the mutant EGFRvIII are major focal points in current concepts of targeted cancer therapy for glioblastoma multiforme (GBM), the most malignant primary brain tumor. The receptors participate in the key processes of tumor cell invasion and tumor-related angiogenesis and their upregulation correlates with the poor prognosis of glioma patients. Glioma cell invasion and increased angiogenesis share mechanisms of the degradation of the extracellular matrix (ECM) through upregulation of ECM-degrading proteases as well as the activation of aberrant signaling pathways. This review describes the role of EGFR and EGFRvIII in those mechanisms which might offer new combined therapeutic approaches targeting EGFR or EGFRvIII together with drug treatments against proteases of the ECM or downstream signaling to increase the inhibitory effects of mono-therapies.

## 1. Introduction

Enhanced angiogenesis and diffuse cell invasion are prominent hallmarks of glioblastoma multiforme (GBM) which add to its lethal character. It is classified as grade IV by the World Health Organization (WHO) classification of tumors of the central nervous system and is diagnosed based on histological parameters like tumor necrosis and, since the current update (2016 WHO Classification of Tumors of the Central Nervous System), on additional molecular features like the status of isocitrate dehydrogenase (IDH). GBMs are divided into two subgroups according to the presence of wildtype or mutated IDH. While IDH mutation occurs in about 10% of cases and corresponds mostly with secondary GBM (resulting from progression of lower grade diffuse glioma), IDH-wildtype is with about 90% of cases the most common type and corresponds frequently with primary or de novo GBM [1]. The patient prognosis is poor; the median survival time is only approximately 14 months after initial diagnosis. Optimized therapy, a combination of radiation and temozolomide, an alkylating agent, increases the percentage of patients alive 2 years after diagnosis but, unfortunately, the tumors usually recur [2].

### 1.1. Glioma Invasion and Angiogenesis

The major reasons that therapies fail are the proliferative and highly invasive nature of the tumor cells and the occurrence of increased angiogenesis. Glioma migration is assisted through the interaction of glioma cells with the neural microenvironment, which can be blood vessels, white matter tracts, brain parenchyma, or the subarachnoid space [3]. The invasion of the cells is a complex process of cell-cell-interaction, extracellular matrix (ECM)-cell interaction, matrix modulation, and cell motility. Tumor growth and invasion needs space, therefore processes to degrade the extracellular environment are incorporated. Through upregulation of proteases such as cysteine proteases, serine proteases, and especially matrix metalloproteases, the tumor cells actively enlarge the space for migration while a parallel activation of aberrant signaling pathways promotes cell motility and proliferation [4]. Angiogenesis, which is the formation of new blood vessels from existing ones, and tumor cell invasion share these mechanisms. The upregulation of angiogenic factors like vascular endothelial growth factor (VEGF), epidermal growth factor (EGF), and fibroblast growth factor (FGF) leads to the secretion of proteases, which degrade the basement membrane and the ECM to enable endothelial cells to proliferate into the surrounding matrix. The inhibition of cell invasion and a reduction of angiogenesis is a favorable aim in glioma therapy to reduce tumor growth and size and to make the tumor mass more accessible to standard therapies and surgery.

### 1.2. The Epidermal Growth Factor and the Mutant EGFRvIII

Epidermal growth factor receptor (EGFR) gene amplification is the most frequent genetic alteration in primary GBM (about 40 percent) and high EGFR expression has been associated with primary human tumors [5]. In addition to gene amplification, a mutant form of EGFR, generally known as EGFRvIII or ΔEGFR, occurs in about 50–60 percent of EGFR-overexpressing GMBs and is exclusively expressed in tumor cells. An in-frame deletion of 267 amino acids from exon 2 to 7 in the extracellular domain of EGFR leads to the inability to bind canonical EGFR ligands. However, EGFRvIII displays constitutive tyrosine kinase activity and mediates persistent intracellular signaling, inefficient ubiquitylation, impaired internalization, and degradation, and therefore escapes downregulation [6,7,8]. EGFRvIII-positive tumors have been associated with poor prognosis and shorter life expectancy and both EGFR and EGFRvIII have been linked to the invasive behavior of glioblastomas together with increased angiogenesis [9,10,11,12,13]. It is also known that EGF promotes tumor angiogenesis, cell motility, and invasion, and that EGFR stimulates cell migration through receptor phosphorylation and subsequent activation of downstream signaling pathways [14,15,16]. However, the cellular and molecular mechanisms behind this and the involvement of the mutant EGFRvIII are still not fully resolved.

There are several treatments available (Table 1), including monoclonal antibodies like cetuximab or small molecule inhibitors like gefitinib, and a vaccination called rindopepimut was even developed to be administered to EGFRvIII-positive tumors [17,18,19]. To complicate things, glioma cells treated with those agents often show resistance mechanisms [20]. They simply overcome the inhibition through increased expression of other growth factors or they switch to different signaling pathways. The latest example of the difficulties in developing effective agents is the failure of a phase III clinical trial of the vaccine rindopepimut, which showed no significant benefit of the vaccine on the overall survival of the patients, despite promising results in a phase II trial [21,22]. A logical consequence to circumvent cell resistance is to apply combination gene therapy and target different molecules or pathways to cover non-overlapping mechanisms and to increase the inhibitory effect. Diverse combination gene therapies are already in clinical trials but there is still need for research to develop novel effective therapeutics. The purpose of this review is to provide current knowledge on how EGFR and EGFRvIII contribute to cell invasion and angiogenesis with a focus on the interaction with ECM-degrading proteases which are involved in matrix modulations and on the activation of transduction signaling pathways. In this context, we discuss the perspective on potential therapeutics and combination gene therapy.

## 2. ECM Remodeling: Induction of Matrix-Degrading Proteases

**Figure 1:**
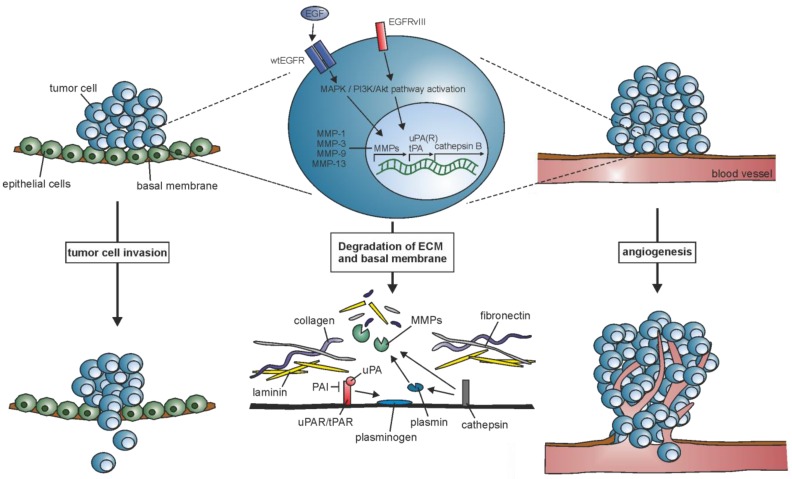
EGFR and EGFRvIII contribute to the upregulation of the extracellular matrix (ECM)-degrading proteases matrix metalloproteases (MMPs), urokinase-type plasminogen activator (uPA), tissue-type plasminogen activator (tPA), and cathepsin B, which leads to degradation of basal membrane components like collagen, laminin, and fibronectin, and facilitate the invasion of tumor cells into the surrounding tissue and blood vessels.

The controlled attachment to or the release from matrix components is inevitable for tumor cell invasion and migration of endothelial cells during angiogenesis (Figure 1). It involves increased proteolytic activity of matrix-degrading proteases which alters cell-cell and cell-ECM interaction to loosen intercellular adhesion and enable migration through the ECM. Matrix metalloproteases (MMPs), serine proteases, and cysteine proteases mediate the cleavage of various, partially shared, substrates such as gelatin, elastin, collagen, plasminogen, and laminin. As their upregulation in glioma correlates with the upregulation of EGFR and EGFRvIII, the proteases might be an interesting target to examine closer.

### 2.1. Matrix Metalloprotease (MMPs)

MMPs are a large group of proteolytic enzymes that degrade ECM proteins in processes associated with morphogenesis, metabolism regulation, and normal tissue turnover. A loss of activity control results in pathological conditions such as arthritis, inflammation, and vascular diseases; a variety of metalloproteases have already been linked to tumor progression in cancer. Among those, gelatinase-A (MMP-2), gelatinase-B (MMP-9), and matrix metalloproteinase-14 (MT1-MMP) are involved in different aspects of the pathophysiology of malignant gliomas [23]. Upregulated expressions are correlated with increased glioma malignancy and the aggressiveness of the tumor. Their expression are both increased in high-grade astrocytomas and their downregulation decreases tumor invasion and tumor growth [23,24].

#### 2.1.1. EGFR and MMPs

In glioblastoma cell line T98G, EGFR upregulates the collagenase MMP-1 via mitogen-activated protein kinase (MAPK) signaling which leads to a decreased cell invasion. An inhibition of EGFR via tyrosine kinase inhibitor AG1478 in turn suppresses EGF-induced MMP-1 expression [25]. The protease MMP-2 is not typically associated with tumor invasion but it is indicated that integrin-mediated EGFR signaling is activated through the direct binding of MMP-2 and PAK4 (a serine/threonine kinase that is involved in the regulation of cell motility, invasion, and growth). As a result, glioma cells develop resistance to anoikis, a form of ECM detachment-induced cell death, increasing their ability to survive in an anchorage-independent manner and therefore facilitating tumor cell invasion and migration [26]. Also, it is already known that EGFR activation promotes MMP-9 expression in different cell types. For example, EGF stimulates migration and MMP-9-dependent invasion in ovarian cancer cells. The process could be impaired through the inhibition of phosphoinositide 3-kinase (PI3K) by LY294002 or wortmannin and MAPK by SB202190 or PD98059, thereby blocking EGF-dependent junction dissolution [27]. The activation of MMP-9 was also correlated with EGFR expression in primary GBM tumors (tumors positive for EGFR, but negative for tumor suppressor protein p53), indicating a link between MMP-9 activation and molecular features of primary GBMs [28]. However, the associated signaling pathways that control MMP-9 remain unclear. Wang et al. discovered the impact of microRNA (miRNA)-181c on MMP-9 in the human glioblastoma cell line A-172. The upregulation of MMP-9 through EGF stimulation could be inhibited by EGFR inhibitor AG1478 or by inhibiting Akt-phosphorylation using LY294002 which blocks the PI3K/Akt pathway. Additionally, overexpression of miRNA-181c downregulates MMP-9 expression at the level of Akt phosphorylation [29].

#### 2.1.2. EGFRvIII and MMPs

The receptor mutant EGFRvIII seems to be mainly involved in upregulation of genes promoting invasive phenotypes like MMP-1 and collagenase-3 (MMP-13), an association shown in athymic mice xenografts [10]. However, this finding stands in contrast to a quantitative proteomics analysis showing only low to no detectible secretion of MMP-1 by EGFRvIII-overexpressing U87 glioma cells [30]. EGFRvIII expression was also strongly correlated with MMP-9 expression, similar to the wild type receptor [28]. The mechanism is still not fully clear, but a regulation through the MAPK/ERK pathway is presumable as it is known that EGFRvIII activates extracellular-signal regulated kinase ERK (1/2) in glioma cells which has been shown to be a direct regulator of MMP-9 [31,32]. In addition to this, a paracrine mechanism driven by EGFRvIII promotes tumor proliferation of EGFR-expressing human glioma cells and stem-like cells (GSCs) and thereby increases tumor heterogeneity and invasiveness [33]. Treatment of EGFRvIII-expressing GSCs with EGFR inhibitors AG1478 or gefitinib promotes invasiveness by suppressing EGFR-mediated transcription factor ID3 expression and subsequently increasing MMP-3 expression [34].

### 2.2. Serine Proteases

The family of serine proteases contains multiple enzymes with a nucleophilic serine in the enzyme active site that cleave peptide bonds in proteins and are ubiquitously expressed in both eukaryotes and prokaryotes. Degradation of the ECM involves the activation of the serine proteases urokinase-type plasminogen activator (uPA) and tissue-type plasminogen activator (tPA) which share plasminogen as the major substrate and specific inhibitors, the plasminogen activator inhibitors (PAI) [4]. uPA and its receptor (uPAR) associate with several members of the integrin family and receptor tyrosine kinases to activate known signaling pathways like MEK-ERK1/2 and JAK-STAT signaling and are frequently expressed at high levels in GBM [35]. Studies showed that they promote glioma invasion and cell growth and protein overexpression was associated with poor prognosis [36].

#### 2.2.1. EGFR and Serine Proteases

PAI-1 is a key regulator of the uPA-uPAR system as its binding with lipoprotein receptor-related protein (LRP) causes the internalization of the ligand-receptor complex [37]. PAI-1 as well as EGFR are highly expressed in grade IV gliomas, and both are poor prognosis markers for overall survival in glioma patients [38]. The in vitro expression of PAI-1, regulated by cytokines and growth factors like EGF, upregulates tPA and uPA in U373 astrocytoma cells followed by a slower increase in PAI-1 [39]. EGFR ligand transforming growth factor (TGFα) upregulates uPA and uPAR, while inhibition via uPAR-specific antibodies decreased the TGFα-induced cell invasiveness [40]. EGFR signaling also increases uPAR-mediated cell invasion downstream of phospholipase Cγ (PLCγ) signaling in human prostate carcinoma and binding of EGF enhances PAI-1 expression via sequential activation of c-Src, protein kinase C-δ, and sphingosine kinase 1 [41,42].

#### 2.2.2. EGFRvIII and Serine Proteases

As of yet, the interaction with EGFRvIII is not well known. However, it was demonstrated that uPAR is required for Stat5b activation downstream of EGFRvIII in EGFRvIII-expressing U87 cells, but can also activate ERK (1/2) even if the cells were treated with tyrosine inhibitors erlotinib and gefitinib [43]. In contrast to wild type EGFR-overexpressing cell lines, PAI-1 was not detected when EGFRvIII was overexpressed [30].

### 2.3. Cysteine Proteases

Cathepsins belong to the papain family, one of six major families of cysteine proteases; cathepsin B is the most studied. It mediates the degradation of the ECM by itself or activates other proteases. Precursor pro-cathepsin D can be activated by several molecules such as cathepsin D and G or uPA and tPA, and cathepsin D in turn can activate uPA, MMPs, and plasmin [4]. Like the MMPs and serine proteases, cathepsin B expression is highly elevated in glioma cells [44].

#### 2.3.1. EGFR and Cysteine Proteases

The mRNA level of cathepsin B correlates with the EGFR expression level [45]. The downregulation of cathepsin B and uPAR decreased pERK levels downstream of EGFR and integrins and increased the expression of p27, indicating an involvement in cell cycle regulation and can therefore induce cell proliferation [46].

#### 2.3.2. EGFRvIII and Cysteine Proteases

The quantitative proteomic analysis, which also revealed the association of EGFRvIII with MMPs, showed that EGFRvIII-overexpressing U87 cells have a stronger invasive profile compared to other alterations in the U87MG cell line and secrete higher levels of cathepsin B [30].

## 3. ECM-Cell Interaction: Activation of Transduction Signaling Pathways

**Figure 2:**
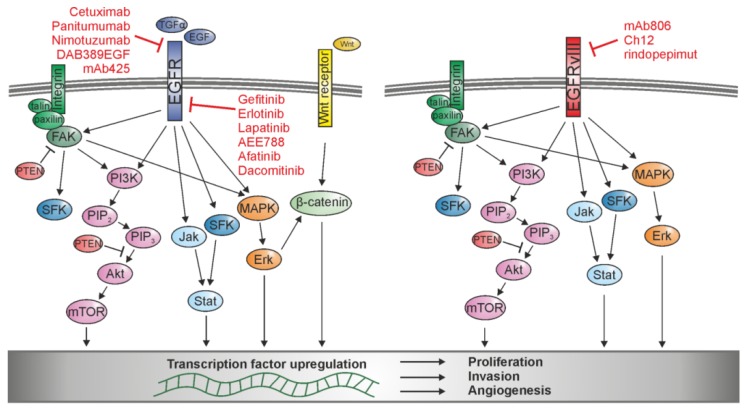
Schematic of signaling pathways activated by EGFR and EGFRvIII and their interaction with integrin and Wnt signaling. Activation upregulates different transcription factors involved in tumor cell proliferation, invasion, and angiogenesis, which can be blocked by various EGFR- and EGFRvIII-specific agents.

The activation of intracellular pathways is the key to pass information from the extracellular environment to the genome of the cell. Through the binding of soluble factors to transmembrane receptors or the interaction between receptors, signals can be transferred and integrated into the cell, increasing or decreasing protein activity and changing and modifying DNA transcription. As already indicated, the preferentially activated pathways by ECM-degrading proteases include the PI3K/Akt, the MAPK, and in some cases the JAK/STAT pathways, which can also be controlled by EGFR and EGFRvIII. Detailed descriptions of the EGFR signaling networks and the deregulated signaling of EGFRvIII in glioma can be found in excellent reviews [47,48]. In this review, we focus specifically on the crosstalk of EGFR- and EGFRvIII with pathways leading to increased tumor invasion and angiogenesis (Figure 2).

### 3.1. Integrin-FAK-ERK Signaling

The integrin family of transmembrane receptors is widely known for their contribution to tumor progression, angiogenesis, and invasion in various tumor types including glioblastomas. Their main function is to link the ECM to the cell’s cytoskeleton but they also take part in specialized cell-cell interactions. It directly binds to ECM glycoproteins and substrates of ECM-degrading proteases such as collagen, fibronectin, and laminin, but also interacts with specific ligands like EGF-like domain-containing protein 7 (EGFL7) to modulate cell adhesion and migration [49]. In addition to the structural role, the clustering of integrin receptors can generate bidirectional signaling to promote cell proliferation, survival, and migration [50,51]. The signaling is transmitted through integrin-associated proteins like talin and paxilin as integrins themselves do not have intrinsic catalytic activity. One of their interaction partners is the focal adhesion kinase FAK, a non-receptor protein-tyrosine kinase which is involved in multiple signaling pathways promoting cell migration and is upregulated in astrocytomas [52,53,54]. FAK binds and activates c-Src kinase (a proto-oncogene which is found to be over-expressed and highly activated in tumors), PI3K, PLCγ, and growth factor receptor-bound protein 2 (Grb2) which links into the MAPK pathway, resulting in the activation of several transcription factors [55,56,57,58].

#### 3.1.1. EGFR in Integrin-FAK-ERK Signaling

The ERK class of MAP kinases can be separately activated by either clustering of integrin receptors and various growth factors but they often collaborate or synergize for a transient activation of the pathway. Phosphorylation of EGFR can not only be induced by the binding of its ligands but also through the interaction of clustered integrin receptors in the absence of EGF, leading to an activation of typical EGFR signaling pathways including the ERK (1/2) pathway [59,60]. This signaling is FAK-dependent, as FAK acts as a bridging protein that links both signals, binding with its N-terminal domain to EGFR [61]. Binding of EGF to EGFR leads to the downregulation of FAK kinase activity in human carcinoma cells overexpressing EGFR. Cells detached from the ECM and showed increased motility and invasion. In the case of reattaching mechanisms, FAK is phosphorylated by an involvement of activated integrin signaling. Therefore, integrin stimulation of FAK promotes adhesion and growth once the tumor cells reattach [62].

#### 3.1.2. EGFRvIII in Integrin-FAK-ERK Signaling

It has been demonstrated that EGFRvIII constitutively activates the MAPK pathway in human glioma cells [32,63]. Substrates of phosphatase and tensin homolog PTEN, a tumor suppressor in many cancer types, includes FAK and inhibits integrin- and growth factor-mediated MAPK signaling [64]. PTEN phosphatase activity suppresses the invasion of EGFRvIII-expressing glioma cells. EGFRvIII could enhance the phosphorylation levels of FAK at Tyr-397 in glioma cells while forming a complex, which correlates with increased catalytic activity of FAK comparable to stimulation by growth factors or integrins. The knockdown of FAK expression reduced the phosphorylation level of ERK (1/2) and inhibited EGFRvIII-induced cell migration in U87ΔEGFR glioma cells [65,66].

### 3.2. SFK Signaling

The nine members of Src family kinase (SFK) are non-receptor tyrosine kinases which are engaged in the modulation of multiple cancer cell processes like cell adhesion, migration, and invasiveness. They are activated by cytokine receptors, receptor protein tyrosine kinases, g-protein coupled receptors, and integrins [67]. Src is frequently upregulated in GBM and the inhibition of SFK activity reduced glioma invasion [68,69,70]. Src increases EGFR-mediated pathway activity in many cancer types and can be activated via FAK-binding which initiates a crosstalk between integrin-FAK signaling and other pathways involved in glioma invasion [71,72,73].

#### 3.2.1. EGFR in SFK Signaling

A recent study suggests and confirms a difference in EGFR and EGFRvIII downstream signaling. Eskilsson et al. found that EGFR was more associated with classical receptor tyrosine kinase signaling and involved in increased cell invasion. Moreover, they could provide evidence that SFK signaling was more specific for EGFRvIII as c-Src was upregulated and constitutively activated in EGFRvIII-expressing GBM cells. Surprisingly, they found those cells to be rather more aggressive in tumor growth and angiogenesis than invasive [74]. In contrast, other findings have demonstrated that Src is involved in enhancing tumor cell invasion and that the interaction is not only exclusive to EGFRvIII but includes interaction with EGFR [71,75]. Both Src and Flyn (both members of SFK) associate with and are phosphorylated by EGFR in glioma cells. Inhibition by the SFK inhibitor dasatinib blocked the EGFR-mediated cell motility in vitro and in vivo [75].

#### 3.2.2. EGFRvIII in SFK Signaling

The kinases Src, Lyn, and Fyn are also expressed in EGFRvIII-expressing glioma cells and promote their tumorigenesis and invasion. As for EGFR-expressing cells, inhibition of these kinases led to decreased invasiveness [75,76]. SFKs also phosphorylate Dock180, a guanine nucleotide exchange factor that activates Rac1 and was found to be involved in the regulation of cell motility and invasion [77]. EGFRvIII stimulates Rac1 signaling through phosphorylation of Dock180 which is mediated by SFKs. Specific shRNA-induced downregulation of SFKs also attenuated EGFRvIII-induced Dock180 activation and decreased glioblastoma cell migration which suggests an EGFRvIII-SFK-Dock180-Rac1 pathway [78].

### 3.3. JAK/STAT Signaling

The Stat family of transcription factors acts as important signaling components and transfers the signal transduction of various cytokines and growth factors like EGF from the extracellular environment to the nucleus. Stat3 is upregulated in many cancer types and promotes immune responses and differentiation as well as cellular transformation, proliferation, and invasion. Upon activation of cell surface receptors through binding of its ligands, the receptor-associated Janus kinase (JAK) and the Src kinase act as connection links between the receptors and Stat3. Stat3 is phosphorylated and translocated into the nucleus where it induces the transcription of multiple genes involved in the regulation of cellular and tissue processes including those that function in cell motility [79].

#### 3.3.1. EGFR in JAK/STAT Signaling

The interaction of EGFR and Stat3 is both physical and functional and described for cytoplasmic EGFR as well as for nuclear EGFR; the level of EGFR correlated significantly with activated Stat3 [80,81]. TWIST is a transcription factor which is required for epithelial-mesenchymal transition (EMT), and elevated levels of TWIST protein correlate with glioma invasion [82]. The binding of EGF to EGFR leads to an activation of Stat3 and subsequently a Stat3-induced increased expression of TWIST [83].

#### 3.3.2. EGFRvIII in JAK/STAT Signaling

Even though EGFR levels show a strong correlation with p-Stat3, the correlation of EGFRvIII with activated Stat3 seems to be even stronger [81]. JAK2 and Stat3 form a complex with EGFRvIII attached to the FAK-Src complex to start an activation loop in EGFRvIII-overexpressing U87 glioma cells which promotes tumor cell invasion. JAK2 inhibitors disrupted this formation leading to a decrease in malignant tumor progression in vivo which was at least partly due to a reduced level of MMP-2 and MMP-9 [84]. Stat3 also associated with EGFRvIII in the nucleus and thereby transformed from a tumor-suppressive protein into an oncogenic protein with the largest effect in a small subset of cells both PTEN-deficient and EGFRvIII-overexpressing [85]. However, how EGFRvIII finds its way into the nucleus remains elusive. To complicate this already complex network of cross-talk between signaling pathways further, it seems that EGFRvIII also cooperates with the wild type receptor as suggested by Fan et al. They showed that even though EGFRvIII is unable to bind constitutive ligands, the binding of EGF does not only lead to a phosphorylation of EGFR but also of the mutant receptor. Phosphorylated EGFRvIII was translocated into the nucleus where it forms a complex and thus enhances the phosphorylation of Stat3 [86].

### 3.4. PI3K/Akt/mTOR Signaling

The PI3K/Akt pathway is known to control cell proliferation, growth, and apoptosis but changes in this pathway can also result in increased metastasis and cell invasion by inhibiting ECM detachment-induced cell death and stimulating MMP secretion [87]. PI3K mediates cell signaling by phosphorylation of phosphatidylinositol-4,5-bisphosphate (PIP_2_) which generates the second messenger PIP_3_. This leads to the activation of Akt, also known as protein kinase B, which transfers the signaling to intracellular targets such as mammalian target of rapamycin (mTOR) and the S6 kinase. The PTEN gene mutation, which is a common occurrence in glioblastoma and which occurs in 30–40% of gliomas, leads to a dysfunctional inhibition of PI3K/Akt signaling by dephosphorylation of PIP3 [88].

#### 3.4.1. EGFR in PI3K/Akt/mTOR Signaling

EGFR activates the PI3K/Akt pathway in both normal and cancer cells and the EGFR gene amplification in glioma cells induces an constitutive activation of PI3K [89]. Inhibition of EGFR through EGFR inhibitors PD153035 or AG1478 leads to the inactivation of the pathway [90]. As a large percentage of gliomas display gene amplification of EGFR or gene mutation of PTEN, the PI3K/Akt pathway is activated in most of them [91]. Evidence has shown that these two pathways not only crosstalk but are also influenced by the evolutionary conserved Notch pathway which is involved in cell fate determination, apoptosis, and cell proliferation. Studies have demonstrated that Akt also induces Notch1 expression in a mouse glioma model and EGFR, conversely, seems to be under the transcriptional control of Notch signaling [92,93,94].

#### 3.4.2. EGFRvIII in PI3K/Akt/mTOR Signaling

In fibroblasts, EGFRvIII activates PI3K more excessively than the wild type receptor [95]. Expression of EGFRvIII increases the activation of Akt through downregulation of the cell cycle inhibitor p27 and enhances cell proliferation [96,97]. In vivo, EGFRvIII has a strong association with the phosphorylation of mTOR and it has been demonstrated that the mutant receptor might be an activator of PI3K in the absence of PTEN loss [98].

### 3.5. Wnt/β-Catenin Signaling

The canonical Wnt/β-catenin pathway has crucial roles in various cellular processes such as cell proliferation as well as cell migration and survival. In the absence of activating ligands, β-catenin, which is located in the cytoplasm, is degraded by a destruction complex consisting of several proteins including the glycogen synthase kinase 3 (GSK3). The binding of Wnt however, leads to a disruption of the destruction complex, followed by a translocation to the nucleus where it serves as a co-activator of transcription factor Tcf/Lef [99]. Aberrations and/or overexpression of key players of the Wnt/β-catenin pathway, like proteins related to the destruction complex or β-catenin itself, have frequently been found in brain tumor cells [100,101,102]. It has been shown that the knockdown of β-catenin inhibited cell proliferation and invasion in glioma cells but the mechanisms behind this are not fully determined [103].

#### 3.5.1. EGFR in Wnt/β-Catenin Signaling

Binding of EGF to EGFR increases activation of Erk which leads to phosphorylation of α-catenin and transactivation of β-catenin [104]. Studies demonstrated further crosstalk between the Wnt/β-catenin pathway and the EGFR signaling pathway since the expression of multiple components of the EGFR pathway such as Stat3, Akt, MMP-2, and MMP-9 were downregulated after β-catenin knockdown [105]. Binding of EGF also induces the endocytosis of E-catherin, a cell adhesion transmembrane protein, resulting in disruption of cell-cell contacts and β-catenin-Tcf/Lef transactivation which contributes to tumor cell invasion [106].

#### 3.5.2. EGFRvIII in Wnt/β-Catenin Signaling

A direct link of EGFRvIII to the Wnt/β-catenin pathway has not been discovered so far. But given the fact that crosstalk between different signaling pathways has been proposed, an indirect involvement of EGFRvIII via constitutive activation of the PI3K/Akt pathway can be assumed. Reports suggested that the activation of GSK-3β, which phosphorylates β-catenin, is regulated by the PI3K/Akt pathway and in glioma, LY294002-induced inhibition of PI3K decreased cell proliferation and cell invasiveness through the downregulation of members of the Wnt/β-catenin pathway, including GSK-3β and β-catenin [107,108].

## 4. Combined Therapy as an Effective Approach for Glioma Treatment

The application of a single target-specific agent as treatment for glioma patients quite often does not produce the desired results, especially in EGFR-or EGFRvIII-specific treatments. The agents on the market for EGFR and EGFRvIII are mostly antibodies binding the extracellular domain or small inhibitory molecules binding the intracellular domain of the protein which block EGFR or EGFRvIII signaling (Figure 2). Although a few of these agents are already approved by the Food and Drug Administration (FDA) for different cancer types, e.g., cetuximab for colorectal cancer and squamous cell carcinoma of the head and neck, gefitinib for non-small cell lung cancer, and erlotinib for non-small cell lung cancer and pancreatic cancer (see Table 1), unfortunately, none are approved for glioma treatment yet. The FDA has designated the monoclonal human antibody nimotuzumab as orphan status for glioma, which means the drug was originally developed to treat diseases affecting fewer than 200,000 people, but also in this case, approval has not yet been granted [109]. Still, efforts are made to develop an effective treatment. Various clinical trials are still ongoing or were recently completed, but in many cases, the desired outcome was not reached. The latest unfortunate examples are a failed phase III clinical trial for rindopepimut with no detected benefit of the vaccine for patient survival and a phase II study of erlotinib and bevacizumab (targeting VEGF) after radiation and temozolomide treatment, which also did not increase the survival of the treated glioma patients [21,110].

This lack of success can be due to different factors including cell resistance against the therapy or heterogeneity of the cells within the tumor. Combination gene therapy raises the possibility to target multiple molecules or pathways to diminish the cell’s ability to upregulate related pathways and circumvent the expected treatment effects. So far, the vast majority of combination studies included EGFR- or EGFRvIII-specific agents together with radiation or broad alkylating reagents like temozolomide. However those therapies might be even more effective when combined with more specific targets. Therefore, in addition to EGFR and EGFRvIII therapy, targeting the responsible matrix-degrading proteases or downstream signaling pathways might prevent the degradation of the ECM and subsequently decrease tumor-related angiogenesis and cell invasion. This could be achieved in more than one way: The direct blocking of the enzyme catalytic activity might prevent the activation of signaling cascades through specific inhibitors, for example tissue inhibitors of the MMPs, the TIMPs. Additionally, one could downregulate the enzymes themselves by downregulating the gene expression by either targeting the preceding signaling pathways, translational factors that activate the expression, or the protein’s mRNA directly. There are various approaches for the inhibition of proteases and members of the signaling pathways available and also already tested. Gene silencing of uPAR, uPA, and MMP-9 by the application of small hairpin RNA (hpRNA) significantly reduced glioma cell invasion and angiogenesis in vitro and direct intratumoral injections of plasmid DNA expressing the hpRNAs regressed pre-established intracranial tumors in nude mice [111]. Moreover, the combinational inhibition of cathepsin B expression and MMP-9 expression via RNA interference lead to reduced glioma tumor growth, invasion, and angiogenesis [112].

The combination gene therapies so far focused rather on the combination of EGFR therapies and targets of the downstream signaling pathways. One well known example is temsirolimus, a specific mTOR inhibitor, which was tested together with erlotinib or with the monoclonal antibody against EGFR, cetuximab, in phase I/II clinical trials [113]. Cetuximab, also called C225, is a chimeric antibody that binds specifically to EGFR consequently preventing the binding of the ligands to the receptor. It is a powerful tool, as it leads not only to an inhibition of the MAPK pathway, but it has also been shown that cetuximab decreases the expression and activity of MMPs like MMP-9, thereby decreasing tumor growth and tumor-related cell invasion in human head and neck squamous carcinoma cell lines and human transitional cell carcinoma [114,115,116,117]. The combination of EGFR inhibition and mTOR signaling pathway inhibition was also tested in GBM with different therapeutics: the application of AEE788, a tyrosine kinase inhibitor for EGFR and in high concentrations for VEGFR together with the application of RAD001, a mTOR inhibitor structurally related to rapamaycin, reduced proliferation and tumor growth in vitro and in vivo by blocking the phosphorylation of Erk and Akt [118]. A recent study has focused on a newly developed antibody against EGFRvIII, called CH12. When used in combination with rapamycin in EGFRvIII-positive and PTEN-negative glioma cells, it effectively inhibited the EGFRvIII downstream signals, including PI3K/Akt and Erk [119]. These studies all confirm that combination gene therapy is a promising approach to effectively increase the inhibitory effect on cell invasion and angiogenesis of mono-therapies against EGFR or EGFRvIII alone.

## 5. Conclusions

The strong ability of glioma cells to invade healthy tissue and to promote tumor-related angiogenesis may limit applicable therapies because of the high risk of tumor recurrence and the capacity of invasive cells to escape the therapy. EGFR and its mutant EGFRvIII have been extensively studied for decades as molecular markers in the context of GBM. In fact, it has been shown that the inhibition of wild type EGFR leads to a less invasive tumor type by switching to an angiogenic state, which shows that even shared mechanisms can be activated in an independent manner [120]. This flexibility of tumor cells, together with the heterogeneity of EGFR expression in general [121], the strong crosstalk and association of the receptor with multiple pathways and invasion- and angiogenic-regulating proteins, and the ability to develop resistance mechanisms are the reasons why mono-therapy of EGFR therapeutics have so far only been partially successful. It ultimately limits the success of therapies such as monoclonal antibody-bases therapies, EGFR- and EGFRvIII-specific small molecule inhibitors, or vaccines, because it is not possible to address all single glioma cells with only one specific treatment. Therefore, new therapies focusing on combination gene therapy with EGFR or EGFRvIII-specific treatments and agents targeting other molecular markers or molecules of collaborating pathways may offer therapeutic advantages. To cover more than one target or more than one mechanism which leads to cell invasion and angiogenesis could increase inhibitory effects. In this review, we focused on the impact of EGFR and EGFRvIII on the upregulation of ECM-degrading proteases and the activation of signaling pathways responsible for increased glioma cell invasion and angiogenesis and the subsequent possibilities to develop effective combined gene therapy. Unfortunately, there are still many unanswered questions and further genetic and molecular investigations are needed to fully reveal the mechanisms behind both glioma cell invasion and angiogenesis. However, early as well as recent studies have shown the promising effect of combination gene therapy, highlighting the potential of future novel therapies to successfully treat human glioma.

## Figures and Tables

**Table 1:** Selection of EGFR and EGFRvIII therapeutics.

Target	Therapy	Class	Targeting also	FDA Approval
EGFR ^1^	Monoclonal antibodies			
	Cetuximab	Mouse-human chimeric antibody	HER1	Colorectal cancer Squamous cell carcinoma of the head and neck
	Nimotuzumab	Human antibody	HER1	Orphan status for glioma Squamous cell carcinoma of the head and neck
	Panitumumab	Human antibody	HER1	Metastatic colorectal cancer
	125 I-Mab 425	Radiolabeled murine antibody	-	N/A ^3^
	Immunotoxins			
	DAB389EGF	EGFR-toxin fusion protein	-	N/A
	Small molecule inhibitors			
	Gefitinib	Anilinoquinazoline-based reversible inhibitor	HER1	Non-small cell lung cancer
	Erlotinib	Anilinoquinazoline-based reversible inhibitor	HER1	Non-small cell lung cancer Pancreatic cancer
	Lapatinib	Thiazolylquinazoline-based reversible inhibitor	HER1/2	HER2+ breast cancer
	Afatinib	Anilinoquinazoline-based reversible inhibitor	HER1/2/4	Metastasized non-small cell lung cancer
	Dacomitinib	Anilinoquinazoline-based reversible inhibitor	HER1/2/4	N/A
	AEE788	Tyrosine kinase inhibitor	VEGFR ^2^, HER1/2, ErbB2	N/A
EGFRvIII	Monoclonal antibodies			
	mAb806	Human antibody	-	N/A
	CH12	Human antibody	-	N/A
	Vaccines			
	Rindopepimut	Peptide vaccination	-	N/A

^1^ EGFR: epidermal growth factor receptor; ^2^ VEGFR: vascular endothelial growth factor receptor; ^3^ N/A: not available.

## Abbreviations

EGFR	Epidermal growth factor receptor
GBM	Glioblastoma multiforme
ECM	Extracellular matrix
IDH	Isocitrate dehydrogenase
VEGF	Vascular endothelial growth factor receptor
EGF	Epidermal growth factor
FGF	Fibroblast growth factor
MMP	Matrix metalloprotease
MAPK	Mitogen-activated protein kinase
PI3K	Phosphoinositide 3-kinase
GSC	Glioma stem-like cells
ERK	Extracellular-signal regulated kinase
uPA	Urokinase-type plasminogen activator
tPA	Tissue-type plasminogen activator
PAI	Plasminogen activator inhibitors
uPAR	Urokinase-type plasminogen activator receptor
PLCγ	Phospholipase Cγ
TGFα	Transforming growth factor
EGFL7	EGF-like domain-containing protein 7
Grb2	Grb2 Growth factor receptor-bound protein 2
PTEN	Phosphatase and tensin homolog
SFK	Src family kinase
JAK	Receptor-associated Janus kinase
PIP_2_	Phosphatidylinositol-4,5-bisphosphate
mTOR	Mammalian target of rapamycin
GSK3	Glycogen synthase kinase 3
